# Targeting Epstein–Barr Virus for Cancer Vaccines: Recent Advances and Future Directions

**DOI:** 10.34133/cancomm.0044

**Published:** 2026-08-21

**Authors:** Guibin Lin, Zibei Chen, Qian Zhong, Cong Sun, Mu-Sheng Zeng

**Affiliations:** State Key Laboratory of Oncology in South China, Guangdong Key Laboratory of Nasopharyngeal Carcinoma Diagnosis and Therapy, Guangdong Provincial Clinical Research Center for Cancer, Sun Yat-sen University Cancer Center, Guangzhou 510060, P. R. China.

In a recent phase I clinical trial (NCT05714748), Peng et al. [1,2] demonstrated that WestGene Biopharma’s mRNA vaccine, WGc-043, achieved disease control rates of 66.67% and 57.14%, respectively, in patients with Epstein–Barr virus (EBV)-positive nasopharyngeal carcinoma (NPC) and natural killer/T cell lymphoma (NKTCL), with a favorable tolerability profile. This encouraging early clinical trial evidence marks a new direction in the treatment of EBV-associated tumors (Fig. 1).

**Fig. 1.**
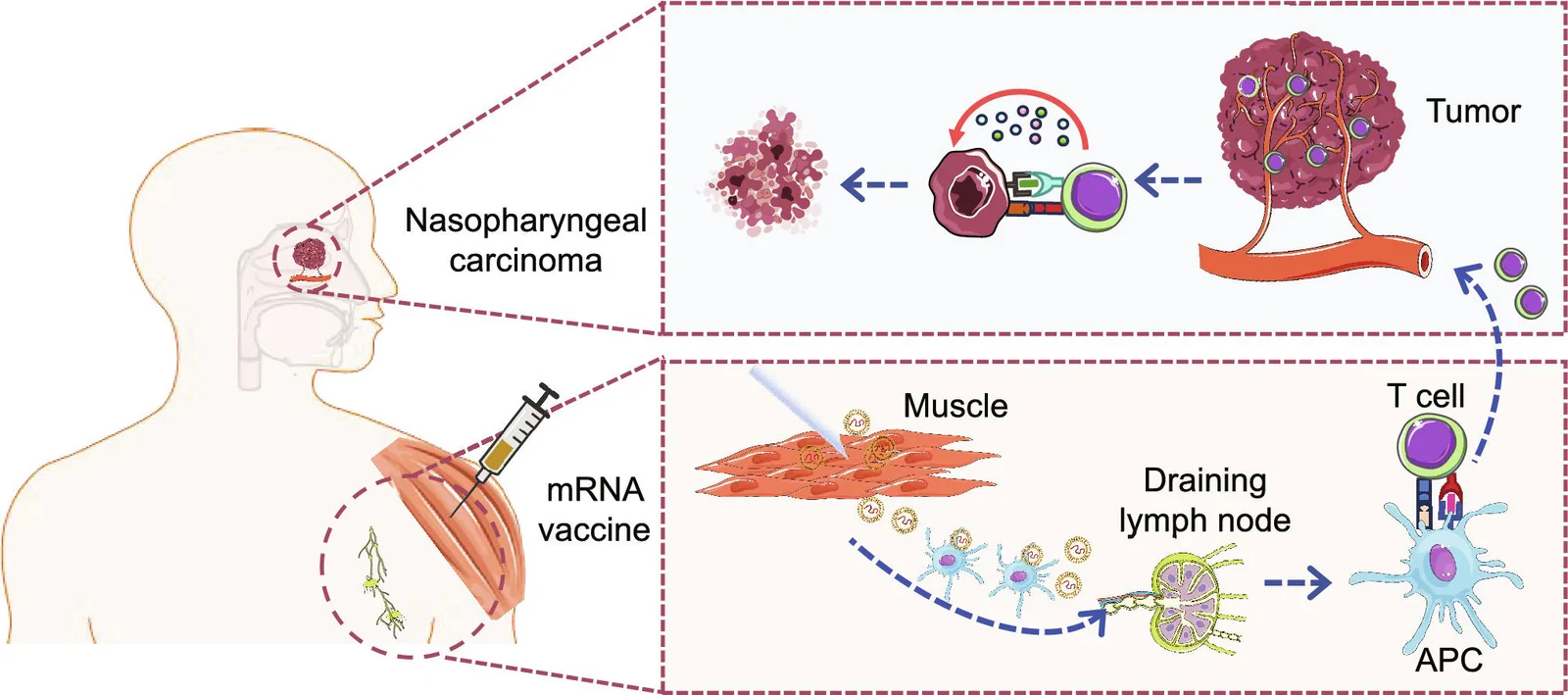
Schematic diagram depicting the mRNA-vaccine-induced immune response against Epstein–Barr virus (EBV)-positive nasopharyngeal carcinoma. Following intramuscular injection of the mRNA vaccine, antigen-presenting cells (APCs) infiltrate the injection site and take up the vaccine. These APCs subsequently migrate to the draining lymph nodes, where they process and present tumor-associated antigens to T cells, leading to T cell activation and clonal expansion. The activated T cells then traffic through the circulatory system to the tumor site to specifically recognize and eliminate nasopharyngeal carcinoma cells.

EBV, the first identified human oncovirus, infects over 90% of the global population. It exhibits dual tropism for epithelial cells and B lymphocytes and is closely linked to malignancies such as NPC [3], NKTCL [4], and Burkitt lymphoma [5]. The establishment of latent infection by EBV in host cells is the primary mechanism of its oncogenesis, during which EBV nuclear antigens (EBNAs) and latent membrane proteins (LMPs) are predominantly expressed. Nearly all cases of nonkeratinizing subtype of NPC, which accounts for more than 95% of NPC cases in endemic regions, are EBV-positive and consistently express EBNA1 and LMP2, along with variable levels of LMP1. Furthermore, EBNA1, LMP1, and LMP2 are highly expressed in NKTCL. Therefore, EBV vaccines targeting latent proteins have emerged as a highly promising therapeutic strategy against EBV-driven malignancies [6]. However, conventional vaccine platforms, including autologous dendritic cells pulsed with LMP2A peptides or LMP2-expressing recombinant adenovirus [7,8], have yet to progress to clinical use despite extensive investigation. The results of this phase I clinical trial highlight the importance of mRNA-based platforms in EBV-targeted vaccine design.

The rise of mRNA vaccine technology has been propelled by the successful development and widespread deployment of severe acute respiratory syndrome coronavirus 2 mRNA vaccines. For instance, an LMP2-targeted mRNA vaccine developed by Guo et al. [9] substantially suppressed tumor growth in an LMP2-expressing mouse model following a 3-dose intravenous regimen. Zhao et al. [10] further reported that mRNA vaccines encoding truncated latent proteins (Trunc-LMP2A, Trunc-EBNA1, and Trunc-EBNA3A), which retain T cell epitope-enriched regions, elicited more potent antigen-specific immune responses than those encoding full-length antigens. These results underscore that both the mRNA vaccine vector and the design of the antigen are crucial for the development of EBV-targeted tumor vaccines. Prophylactic EBV vaccines primarily aim to prevent EBV infection through the induction of neutralizing antibody responses against viral entry glycoproteins. In contrast, therapeutic EBV vaccines are designed to stimulate potent EBV-specific cellular immunity, particularly cytotoxic T cell responses, against latent viral antigens expressed by infected or malignant cells. These mRNA platforms are therefore attractive owing to their capacity to rapidly encode multiple antigens, induce both humoral and cellular immune responses, and avoid genomic integration.

To evaluate the immunogenicity, safety, and tolerability of WGc-043, Peng et al. [1,2] administered it via 5 intramuscular injections, followed by optional extension of giving the same dose of WGc-043 mRNA vaccine monthly for the treatment of EBV-associated tumors. The therapeutic efficacy was assessed by analyzing immune responses in patient peripheral blood and tumor tissue samples, and safety profiles were determined through adverse event monitoring. Among the 12 patients with EBV-positive NPC, 2 achieved partial response, and 6 had stable disease. Notably, 91.7% of the subjects exhibited a substantial reduction in plasma EBV DNA levels postadministration. In the 7 patients with EBV-positive NKTCL, partial response was observed in 3 patients and stable disease in 1 patient. Although WGc-043 injection showed encouraging preliminary activity in patients with EBV-positive NPC and NKTCL, its therapeutic potential remains to be fully evaluated given the limited number of patients and phase I design of the trial. In addition, Moderna Inc. has developed mRNA vaccines targeting EBV (mRNA-1189 and mRNA-1195). mRNA-1189 encodes viral envelope glycoproteins, including gp350, gp42, and the gH/gL complex, which are located on the viral surface, aiming to prevent infectious mononucleosis. mRNA-1195 contains glycoproteins and latent antigens and is designed to simultaneously induce antibody and T cell responses to combat posttransplant lymphoproliferative disorder and multiple sclerosis. These 2 candidate mRNA vaccines are still in the process of clinical investigation.

Despite the promise of EBV-targeted mRNA vaccines in oncology, several challenges persist. First, because current mRNA vaccines encode only a limited repertoire of latent EBV antigens, tumor heterogeneity and variable antigen expression may promote immune escape through the selective expansion of antigen-negative tumor subclones. Second, monotherapy is unlikely to achieve complete tumor eradication because of the short duration of antigen expression, limited intratumoral T cell infiltration, and the immunosuppressive tumor microenvironment, thereby necessitating synergistic combination strategies. Third, current mRNA vaccines are usually delivered by lipid nanoparticles. This nontargeted delivery carries the risk of drug accumulation in the liver and off-target antigen production. Fourth, repeated administration of therapeutic mRNA vaccines may be associated with potential safety concerns, including excessive innate immune activation and rare inflammatory cardiac adverse events such as myocarditis. Therefore, the long-term safety profile of repeated mRNA vaccination in oncology settings warrants further investigation. Finally, most clinical evidence remains preliminary, underscoring the need for larger randomized trials to confirm efficacy and long-term safety across diverse populations.

Future efforts should be directed along several key axes. In antigen design, EBV-targeted mRNA vaccines should expand beyond single antigens to include broader repertoires, while also optimizing sequences for enhanced expression and presentation and accounting for intratumoral EBV antigen mutations. Regarding mRNA vaccine delivery, there is a need to engineer more efficient, targeted, and safer delivery vehicles. Therapeutically, combining EBV-targeted mRNA vaccines with other immunomodulators (e.g., checkpoint inhibitors) or conventional therapies represents a rational strategy to synergistically enhance antitumor immunity and overcome resistance.

In conclusion, mRNA vaccines represent a transformative frontier in managing EBV-associated cancers. While current preclinical and early clinical findings are encouraging, the available evidence remains preliminary, and substantial challenges related to tumor heterogeneity, delivery efficiency, immune durability, safety, and clinical translation must still be addressed before broader clinical application can be achieved.

## Ethical Approval

Not applicable.

## Data Availability

Not applicable.
